# Using defects to store energy in materials – a computational study

**DOI:** 10.1038/s41598-017-01434-8

**Published:** 2017-06-13

**Authors:** I-Te Lu, Marco Bernardi

**Affiliations:** Department of Applied Physics and Materials Science, California Institute of Technology, Pasadena, CA 91125 USA

## Abstract

Energy storage occurs in a variety of physical and chemical processes. In particular, defects in materials can be regarded as energy storage units since they are long-lived and require energy to be formed. Here, we investigate energy storage in non-equilibrium populations of materials defects, such as those generated by bombardment or irradiation. We first estimate upper limits and trends for energy storage using defects. First-principles calculations are then employed to compute the stored energy in the most promising elemental materials, including tungsten, silicon, graphite, diamond and graphene, for point defects such as vacancies, interstitials and Frenkel pairs. We find that defect concentrations achievable experimentally (~0.1–1 at.%) can store large energies per volume and weight, up to ~5 MJ/L and 1.5 MJ/kg for covalent materials. Engineering challenges and proof-of-concept devices for storing and releasing energy with defects are discussed. Our work demonstrates the potential of storing energy using defects in materials.

## Introduction

Defects in solids play a central role in energy applications. Point defects can catalyze chemical reactions^1^, control the efficiency of light emission^2–4^, and tune the electrical^5, 6^ and thermal^7–9^ properties of materials. Defects in crystalline materials require energy to be generated, and as such they can be regarded as excited states of the crystal. Point defects exist spontaneously in solids due to configurational entropy^10^ and thus the defect formation energy cannot be extracted from the material in thermal equilibrium, since this would violate the second law of thermodynamics.

However, non-equilibrium defects generated by neutron^11^, ion^12^ or electron^13, 14^ bombardment, or through laser irradiation^15^, can store part of the energy transferred to the material by the incoming particles. This stored energy associated with defects can reach surprisingly large densities. For example, the Wigner energy^16, 17^ stored in graphite defects under neutron bombardment can reach values as high as 2.7 MJ/kg, and lead to bursts of released energies intense enough to cause fires, as it occurred in the notorious Windscale nuclear accident^18^.

Defects can be rather long-lived, with lifetimes of up to weeks at low temperature^19^. In addition, since the timescale for defect recombination is highly temperature dependent, favorable conditions to maintain non-equilibrium defect populations can be engineered. In spite of the potentially large stored energy densities and long lifetimes, defects are not traditionally regarded as an approach for energy storage. In particular, the idea of intentionally generating defects in a material to capture energy at one time, and release this energy at a later time (e.g., by annealing the material) has not been explored in detail.

Here, we investigate energy storage in materials defects. We obtain trends and upper bounds for energy storage with defects, and carry out first-principles calculations of the most promising materials. Combining density functional theory (DFT) calculations with previously measured and computed data, we obtain the energy per unit volume and weight stored by a range of defects, including vacancies, interstitials and Frenkel pairs in bulk elemental materials, and Stone-Wales defects in graphene. For a reference point defect concentration of 1 at.%, we find that metals can store energy densities of 0.2–1.1 MJ/L and specific energies of 0.07–0.5 MJ/kg, while covalently bonded materials such as graphite and diamond can store energies as high as 2.1–4.9 MJ/L and 0.9–1.4 MJ/kg, which are comparable with the energy stored by state-of-the-art batteries and supercapacitors^20, 21^. Even a small and readily achievable defect concentration of 0.1 at.% can store energy densities of up to ~0.5 MJ/L and ~0.15 MJ/kg. Practical aspects, devices, and engineering challenges for storing and releasing energy using defects are discussed. The main challenges for defect energy storage appear to be practical rather than conceptual. We identify four main challenges: (1) Realizing simple and inexpensive techniques to generate defects; (2) Initiating defect recombination with minimal waste of heat into the environment; (3) Finding promising materials with high stored energies that are also mechanically and chemically stable during the defect generation and recombination processes; (4) Reversibly storing and releasing energy using defects. In spite of these engineering challenges, our work demonstrates that defects are a possible new approach for energy storage.

## Results

### Stored Energy Estimates and Upper Bounds

**Figure 1:**
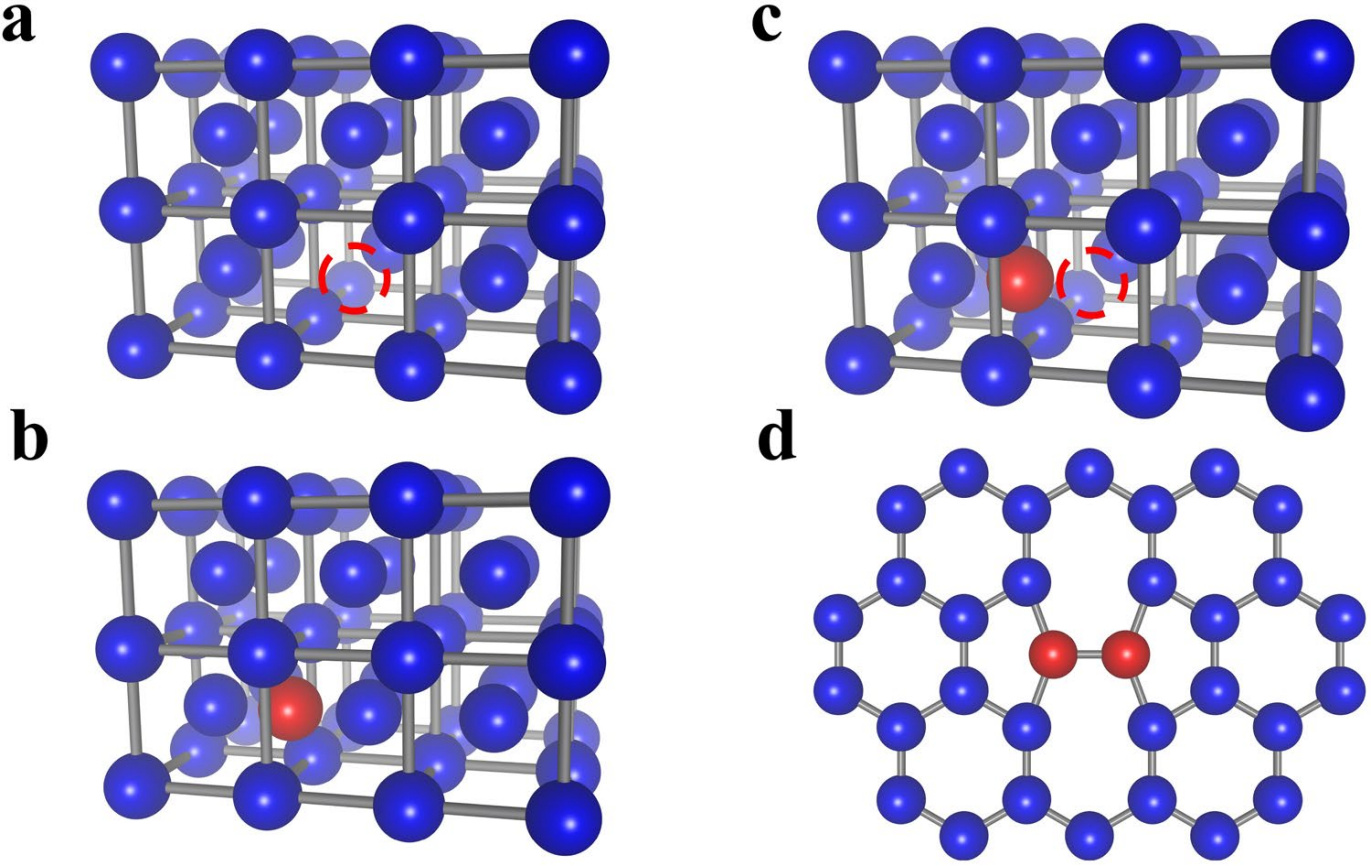
Defects considered in this work, including (**a**) vacancy, (**b**) interstitial and (**c**) Frenkel pair defects in a bulk crystal, and (**d**) Stone-Wales (SW) defects in graphene. Interstitial and SW defects are indicated by red atoms, and vacancies by red empty circles.

Point defects such as vacancies and interstitials (Fig. 1a,b) are commonly found in materials at equilibrium. In this work, we additionally consider defects typically formed upon bombardment or irradiation, including Frenkel pair (FP) defects (Fig. 1c), which consist of a pair of interacting vacancy and interstitial, and Stone-Wales (SW) defects (Fig. 1d) in graphene. The design space of materials and types of defects with potential for energy storage is vast. We first estimate the energy stored in these defects for different elemental materials, expressing the results in terms of the energy density and specific energy, defined here and in the following as the stored energy per unit volume and weight, respectively. We then focus on the most promising cases by carrying out first-principles DFT calculations.

Typical defect formation energies are of order 0.5–8 eV for vacancies and 2–10 eV for interstitials in most materials^22^. These values can be combined to estimate the formation energy of FPs, *E*_*FP*_, which is not readily available in the literature. The vacancy and interstitial in a FP attract each other by lowering the strain field, so that the formation energy of an isolated FP (IFP), consisting of a vacancy and interstitial infinitely far apart, overestimates *E*_*FP*_ by the vacancy-interstitial attractive energy. The IFP formation energy, obtained as the sum of the vacancy and interstitial formation energies, provides an upper limit to *E*_*FP*_ of approximately 2.5–13 eV for most materials. Using typical atomic densities, we estimate for a reference FP concentration of 1 at.% (see below) a stored energy density of ~0.05–2.5 MJ/L and a specific energy of 0.005–0.25 MJ/kg. These values establish an upper bound of the energy stored by a 1 at.% concentration of point defects such as  vacancies, interstitials, and FPs.

**Figure 2:**
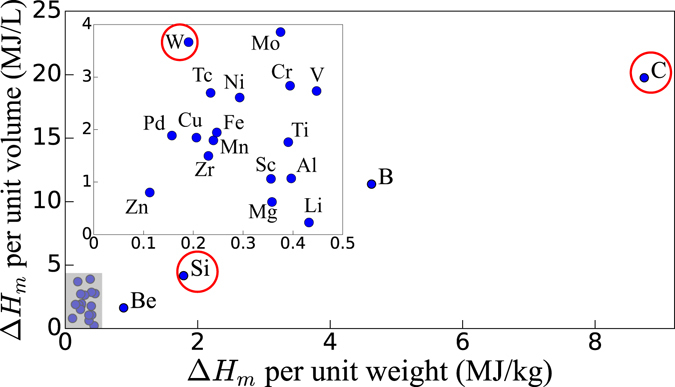
Heat of melting Δ*H*_*m*_ per unit volume and weight for a range of elemental materials; the inset zooms in the gray shaded region. Here, C is the graphite allotrope. Some covalent materials (e.g., B, C and Si) possess heat of melting values significantly higher than all other elements. Red circles indicate materials selected for first-principles calculations.

The heat of melting Δ*H*_*m*_ can alternatively be taken as an upper bound of the energy stored by defects. Materials bombarded with ions or neutrons, or irradiated with intense light, become disordered and undergo a transition to an amorphous state above a threshold concentration of irradiation-induced defects^23, 24^. Upper bounds of the energy stored by defects can be obtained using Δ*H*_*m*_ to approximate the energy of the amorphous state with respect to the pristine crystal. Figure 2 shows the heat of melting per unit volume and weight, taken from the literature^25^, for a range of metals and covalent materials. We find values of order 0.05–4 MJ/L and 0.05–1 MJ/kg for most materials, which are slightly higher than the estimates obtained above for a 1 at.% concentration of FPs due to the fact that amorphization typically occurs for defect concentrations of up to 10 at.%. The upper bounds derived in this section are comparable to the energy density of state-of-the-art Li ion batteries (roughly 0.8–1.75 MJ/L)^20, 26^, a physically sound result since both batteries and defects store energy by locally rearranging bonds in materials. Practical matters aside, our estimates show that defects can in principle store energy densities as high as those of established energy storage technologies.

### Materials Selection and Stored Energy Computations

Materials with high defect formation energies are desirable to maximize the stored energy density. The heat of melting and cohesive energy, both of which correlate with bond strength and thus with defect formation energy, are shown respectively in Fig. 2 and Fig. S1 (see Supplementary Information) for a range of metallic and covalent elements. Due to the relative weakness of the metallic bond, both the heat of melting and the cohesive energy per unit volume of metals are lower than those of covalent materials. The higher densities in metals further contribute to lower these values per unit weight. Exceptions include Li and Be due to their low density, and W due to its strong and partially covalent bond. These elements possess the largest heat of melting and cohesive energy per unit weight (Li, Be) and volume (W) among metals, and are thus promising for energy storage using defects. Among covalently bonded materials, lightweight elements such as B, C and Si provide the largest heat of melting and cohesive energy densities, while graphite exhibits the largest energies per unit volume and weight of all materials, consistent with its known ability to release large amounts of energy stored in defects^18^.

Using these data, we select promising elemental materials for energy storage in defects, including a metal (tungsten) and four covalent materials: diamond, graphite, silicon and graphene. We employ DFT calculations (see Supplementary Information for computational details, Fig. S2–S6 for defect configurations, and Fig. S7 and Table S1–S2 for defect formation energy results) to compute the defect formation energy *E*_*F*_ of vacancies, interstitials, and FP in these materials. The stored energy density *E*:

1 $$E={E}_{F}\times {C}_{NE},$$

is computed using a reference non-equilibrium concentration of defects *C*_*NE*_ = 1 at.%, which approximates the highest defect concentrations achievable in most materials. We focus our discussion on FPs in bulk materials and the SW defect in graphene, both because these defects are commonly generated by irradiation and because their stored energy is an upper bound for other point defects. Note however that using the data in Table S1 (our DFT computations) and Table S3 (literature formation energies) given in the Supplementary Information, stored energies can also be obtained with eq. 1 for arbitrary concentrations of vacancies, interstitials, and FPs.

**Figure 3:**
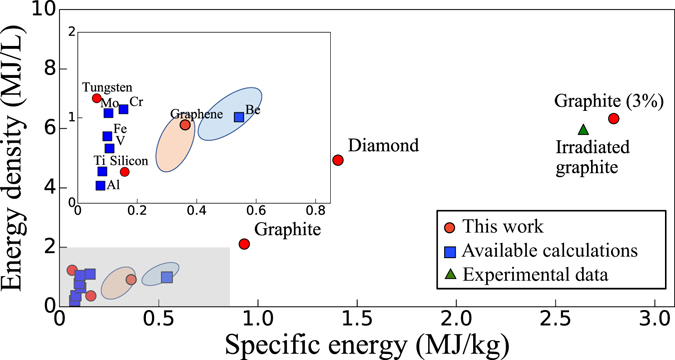
The energy density and specific energy stored in materials defects, shown here for a 1 at.% concentration of FPs in bulk materials and SW defects in graphene. The inset zooms into the gray shaded region. The FP (or SW) formation energies computed here (red circles) are taken from Table S1 and those obtained from the literature (blue squares) are given in Table S3 (see Supplementary Information). The experimental^27–29^ (green triangle) and computed stored energy for a 3 at.% concentration of FPs in irradiated graphite are also shown. For comparison, we plot the range of stored energies in Li-ion and Ni-MH batteries^20^.

Figure 3 shows the stored energy density and specific energy for a range of materials, obtained using the FP (for graphene, the SW) formation energies computed with DFT for the most promising elemental materials (red circles), as given in Table S1, as well as data available in the literature (blue circles) for other elements (see Table S3). For metals with a 1 at.% concentration of FPs, we obtain stored energy densities of 0.2–1.1 MJ/L, and relatively small specific energy values of 0.07–0.5 MJ/kg. For covalent materials with a 1 at.% concentration of FPs, we find greater energy density (0.4–4.9 MJ/L) and specific energy (0.15–1.4 MJ/kg) values than in metals. In particular, graphite and diamond achieve greater energy densities and specific energies than those of any metal. These computed values for a 1 at.% concentration of FPs are a factor of 2–10 lower than our upper bound estimates obtained using the heat of melting. In some materials, the concentration of defects can be greater than our chosen 1 at.% reference; for example, the FP concentration in graphite can be as large as ~3 at.%^27, 28^ at room temperature. Our computed energy density (6.2 MJ/L) and specific energy (2.8 MJ/kg) for graphite with such a 3 at.% FP concentration are in very good agreement, respectively, with the experimental values of 6.0 MJ/L and 2.6 MJ/kg measured in heavily irradiated graphite^29^ (see the green triangle in Fig. 3). This result validates our approach and shows that first-principles calculations can accurately compute the energy stored in defects.

The energy densities and specific energies of defects are compared in Fig. 3 with those of established energy storage technologies such as batteries and supercapacitors. Note that practically achievable values, as opposed to ideal limits, are employed for this comparison. In metals, the specific energies of 0.07–0.5 MJ/kg for a 1 at.% FP concentration are greater than those of supercapacitors (~0.035 MJ/kg)^30^, and their energy densities are comparable with nickel-metal hydride (Ni-MH) and Li-ion batteries. Covalent materials such as graphite and diamond with a 1 at.% FP concentration can achieve stored energies roughly twice greater than those of Li-ion batteries (up to ~1.75 MJ/L and ~0.75 MJ/kg, respectively)^26^. This result is striking given that defects in materials are not commonly regarded as a technology for energy storage. The stored energy values for 0.1–1 at.% defect concentrations, which can be achieved routinely with bombardment or irradiation, show that defects in materials, if properly engineered, may achieve stored energies comparable with those of state-of-the-art technologies.

Lastly, the choice of a reference non-equilibrium concentration in eq. 1 is non-trivial. Defects with a wide range of concentrations can be prepared experimentally, including FP concentrations of 0.2–0.6 at.% in most metals at low temperature^31^ and up to 3 at.% in graphite at room temperature^27^. Our choice of 1 at.% represents a practically achievable limit in most materials. Yet, defect concentrations as high as ~10 at.% have been recently achieved in thin crystals of MoS_2_^32^, with potential for stored energies much greater than those reported here.

## Discussion

**Figure 4:**
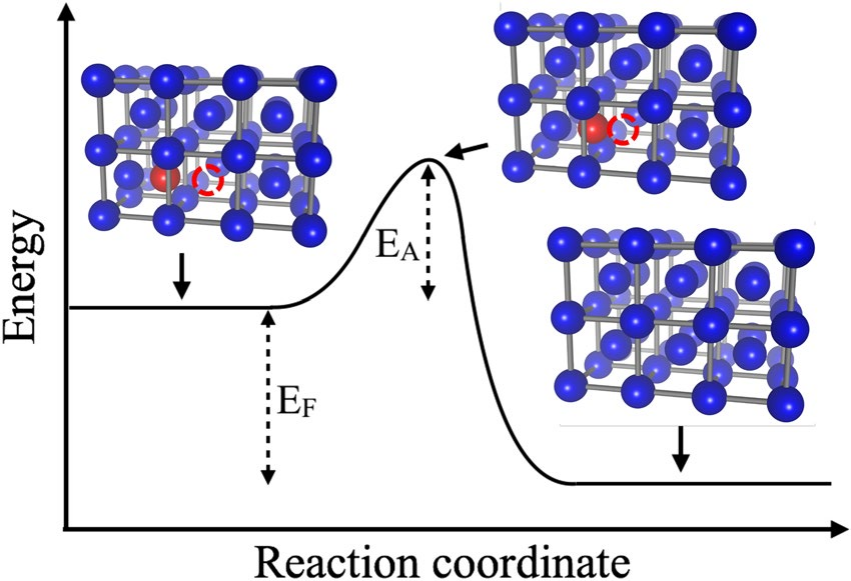
Energetics of defect formation and recombination, shown for a FP defect consisting of a vacancy (red empty circle) and interstitial (red atom) pair. The states involved in the recombination reaction are the FP defect (left), transition state (center) and pristine lattice (right). The FP formation energy *E*_*F*_ and the activation barrier *E*_*A*_ are also shown. The energy *E*_*A*_ needs to be provided for FP recombination.

While feasible in principle, reversibly storing energy in materials defects poses significant practical challenges. The kinetic events associated with energy storage and release in defects are shown in Fig. 4. Defect generation stores an amount of energy per defect equal to the formation energy *E*_*F*_. While defect recombination releases this energy in the form of heat, it requires activation over the energy barrier *E*_*A*_ between the defect and transition state in the recombination reaction (Fig. 4).

We discuss possible approaches for storing and releasing energy in defects, as well as their efficiency and practical realization. Typical methods employed to generate defects include bombardment with energetic electrons, ions, atoms, thermal or fast neutrons, as well as plasma processing^32^. The concentration of generated defects typically increases with the dose of incoming particles, and decreases for increasing temperature and defect recombination rates. A variety of defect types and concentrations can be generated through these bombardment methods, with greatly varying energy cost among different techniques. For example, electrons and ions need to be accelerated – at an energy cost – to generate defects; neutrons, on the other hand, are spontaneously available in nuclear plants or in the presence of nuclear decay processes. An efficiency for energy storage *η*_*S*_ can be defined as the ratio of the formation energy of the defects to the energy necessary to generate them. Beyond an optimal dose, the generated defects recombine and ultimately attain a steady-state concentration, so that irradiating the material beyond this optimal dose decreases *η*_*S*_. To go beyond these general considerations, the detailed value of *η*_*S*_ needs to be obtained on a case-by-case basis.

Optimal methods for releasing the stored energy are those able to activate the defects above the barrier *E*_*A*_ with minimal energy waste. In defining an efficiency for energy release *η*_*R*_, it is important to note that while heat is required to overcome the activation barrier for recombination, *this heat is not lost*, but rather it is returned to the environment when the defect recombines. From a microscopic viewpoint, following activation to the transient state by providing an energy *E*_*A*_, the energy *E*_*A*_ + *E*_*F*_ is returned, rather than just *E*_*F*_. Defect energy release should thus be regarded as an approach for boosting, as opposed to replacing, heating from a conventional source. The energy release efficiency can be defined as:

$${\eta }_{R}=\frac{{\rm{released}}\,{\rm{defect}}\,{\rm{energy}}+{\rm{energy}}\,{\rm{input}}}{{\rm{energy}}\,{\rm{input}}}$$

where the energy input is the energy spent to heat up the material and overcome the recombination barrier, and the efficiency is above unity, *η*_*R*_ > 1.

**Figure 5:**
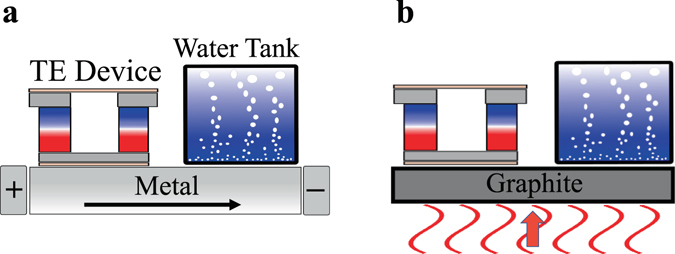
Proof-of-concept device designs for releasing the energy stored in defects. (**a**) Joule heating of a metal using an electric current, and (**b**) exposing the material (here, graphite) to radiant heat generated by absorbing solar energy. In both examples, the released energy can be converted to electricity via a thermoelectric (TE) device, or employed to run a heat engine or to generate steam by heating up a water tank.

Both traditional and unconventional approaches are available to activate defect recombination. The conventional approach of heating the material in a furnace is viable but energetically costly, while Joule heating in metals and transient annealing^33^ in semiconductors are two approaches providing more localized heating. A possible design implementing Joule heating for energy release is shown in Fig. 5a, where an electric current is applied to a defect-containing metal or doped semiconductor. The energy dissipated by the current activates the defects, which in turn provide additional heat upon recombination. Note that the energy from the current is not lost, but simply borrowed to overcome the transition state and then returned upon recombination. For semiconductors, transient heating methods using lasers, electron or ion beams, and other radiant sources have been widely employed as energy-efficient alternatives to furnace heating^33^. Radiant heating (Fig. 5b) is also a possible approach to induce defect recombination.

For the two proof-of-concept devices proposed in Fig. 5, the source could be renewable solar energy – photovoltaics for Joule heating, and solar thermal energy for direct or radiant heating of the material. These devices could be employed to generate steam, or to run a heat engine or thermoelectric device to make electricity. Positive-feedback heating is also possible with defects, whereby the energy released by some defects activates recombination of more defects, a feasible strategy provided that thermal runaway, as in the Windscale accident, is avoided. The temperature change due to defect recombination, together with the heat release rate and duration, depend on the heat capacities of both the material hosting the defects and its surroundings. Strategies to optimize heat release thus need to be evaluated on a case-by-case basis.

Defect recombination is an energy release process with peculiar properties. Not all the stored energy is released at once in a typical annealing process, since defects with significant concentrations interact and form aggregates, clusters and voids^34^. Due to defect aggregation, multiple energy release steps are commonly observed experimentally (for example, in irradiated graphite^35^) rather than just one energy release event. To release all the stored energy at once, the defect aggregation problem would need to be addressed.

The power density per unit volume (W/L units) or weight (W/kg units) are important quantities that vary widely among storage technologies. The power density released from defect recombination can be computed if the defect lifetime is known. For example, graphite with a 1 at.% concentration of FPs stores a specific energy of ~1 MJ/kg and an energy density of ~2 MJ/L. Using a conservative estimate of the room temperature FP lifetime in graphite of a few minutes to a few hours, and assuming that all the stored energy can be released at once, then the power density would be of order 10^3^–10^4^ W/L and 10^3^–10^4^ W/kg, and thus potentially as high as in supercapacitors^36^. Since the atomic diffusion rate in solids follows an Arrhenius law, the defect recombination lifetime varies exponentially^19^, so that power density released by defects is widely tunable, perhaps a unique feature among energy storage technologies.

The two main quantities of interest to engineer heat release from defects are the activation energy for defect recombination and the defect lifetime. The activation energy for FP recombination is typically in the 0.5–2 eV range and is available in the literature for several materials^34, 37, 38^, including metals^22, 39^ where vacancy or interstitial migration is the only barrier for FP recombination. The defect lifetimes, on the other hand, are still challenging to compute or measure and deserve further investigation.

Based on the data presented in this work, we believe that defects have the potential to become a new niche in energy storage research, with possible applications to heating, catalysis, metal fuels^40^, combustion and space shuttles, among others. Interestingly, defects in crystalline materials can be seen as part of a broad new family of emerging approaches for energy storage based on changes in atomic bonding in molecules and solids. One such approach employs molecular solar thermal fuels such as azobenzene to reversibly store solar energy in molecular bonds^41–43^ by converting the lower energy *trans* to the metastable *cis* isomer; the stored energy can be released by catalyzing the isomerization to the *trans* ground state^41–43^. The parallel with defects in solids is apparent if one regards the *trans*–*cis* conformation change as the creation of a defect in a molecule. In this respect, defect energy storage in solid-state materials generalizes the idea of storing energy in molecular bonds.

## Conclusion

In summary, we demonstrate that defects in materials can store energy with densities as high as those of widely employed storage technologies. Engineering reliable and efficient energy storage devices based on defects remains an open challenge. While our work focuses on elemental materials, a wide range of metal alloys and covalent or ionic compounds still need to be explored, with potential to achieve higher stored energies than those found here, especially in alloys and compounds with strong inter-atomic bonds. Ideal materials and defect combinations could be searched, for example, with high-throughput materials genome approaches^44^. The criteria derived here show that lightweight materials with strong bonds and large defect formation energies are ideal to achieve high stored energy densities. These results will be useful guidelines to identify novel materials for energy storage using defects.

## Methods

We obtain the defect formation energies *E*_*F*_ by computing the total energy *E*_0_ of a pristine unit cell containing *N*_0_ atoms and no defects, and the total energy *E*_*D*_ of a supercell containing N atoms and one defect:

2 $${E}_{F}={E}_{D}-(\frac{N}{{N}_{0}})\times {E}_{0}$$

We carry out DFT calculations within the local-density approximation (LDA)^45^ using the QUANTUM ESPRESSO code^46^. Supercells with increasing number of atoms (*N*) are employed to converge the defect formation energy by extrapolation to the *N* → ∞ limit. We use ultrasoft pseudopotentials^47^ to describe the core-valence electron interactions, together with a plane-wave basis set with kinetic energy cutoffs in the 40–60 Ry range and Monkhorst-Pack **k**-point grids of up to 30 × 30 × 30 for bulk materials and 60 × 60 × 1 for graphene. The kinetic energy cutoff and **k**-point grids are chosen using a 10 meV/atom convergence criterion on the total energy. Experimental lattice constants are employed in the calculations. Upon relaxation, the residual atomic forces in the relaxed structures are less than 25 meV/Å. We choose to employ the LDA approximation to achieve a trade-off between computational cost and accuracy for the large (up to ~500 atoms) unit cells with defects employed here. For tungsten we use the Perdew-Wang 91 (PW91) exchange-correlation functional^48, 49^ in combination with the DFT relaxed lattice constant. These choices are due to the large difference in the defect formation energy between the LDA and experimental results in tungsten. Additional computational details on the structures and **k**-point grids are provided in the Supplementary Information.

## Electronic supplementary material

Supplementary Information
