# Supplementary material for: Using defects to store energy in materials – a computational study
Source: Sci Rep. 2017 Jun 13;7:3403. doi: 10.1038/s41598-017-01434-8 (PMC5469865; doi:10.1038/s41598-017-01434-8)
Supplement: Supplementary file 1 — Supplementary Information [file 41598_2017_1434_MOESM1_ESM.pdf]

Supplementary Information for:

Using defects to store energy in materials – a computational study

I-Te Lu and Marco Bernardi*

Department of Applied Physics and Materials Science, Steele Laboratory,

California Institute of Technology, Pasadena, CA 91125, USA

*Corresponding author. Email: bmarco@caltech.edu

COHESIVE ENERGY DENSITY TRENDS

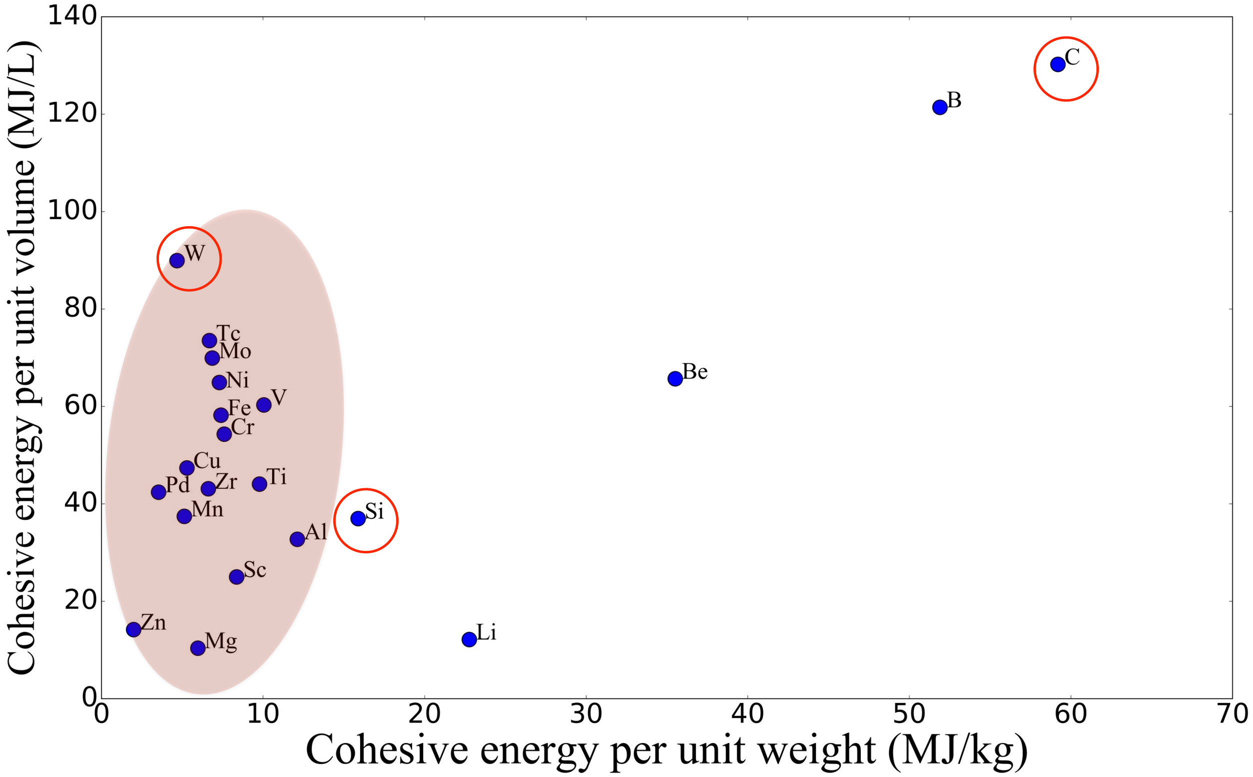

Figure S1. The cohesive energies per unit volume (MJ/L units) and weight (MJ/kg units), taken from the literature[1], are shown for the same materials as those in Fig. 2 of the main text. The trends for the cohesive energy across the periodic table resemble those for the latent heat per unit volume and weight shown in Fig. 2 of the main text. Here, C is the graphite allotrope, and red circles are used for the most promising materials for energy storage in defects, for which first-principles calculations are carried out in this work.

DEFECT FORMATION ENERGY CALCULATIONS

The defect formation energy E_F is computed under the assumption that defects do not interact with each other, so that E_F is the energy to form an isolated defect. Figure S7 shows the convergence of E_F, achieved by employing supercells with a single defect and progressively increasing the supercell size to remove spurious image interactions[2]. The formation energy is then obtained by extrapolating the results to the $N \to \infty$ limit. These extrapolated results, used in Figure 3 of the main text, are given below in Table S1. Note that since the defects studied here are neutral (i.e., not charged), no additional corrections due to the periodic boundary conditions are necessary. Next, we provide additional details on the calculations carried out in this work.

Graphene: we use a hexagonal 2-atom unit cell with an experimental lattice constant of 2.46 Å and a 60×60×1 **k**-point grid. For the $n \times n \times 1$ supercells with the Stone-Wales defect, we use **k**-point grids of 10×10×1, 9×9×1, 8×8×1, and 6×6×1 for supercells with $n = 6, 7, 8,$ and 10, respectively. A 20 Å vacuum in the layer-normal direction is included in the simulation cell in all cases. The relaxed structure is shown in Fig. S2.

Graphite: we use a hexagonal 4-atom unit cell with experimental lattice constants of a = 2.46 Å and c = 6.708 Å. The **k**-point grid for the unit cell is 60×60×8. For the vacancy, we use the following $n \times n \times 1$ supercells: n=4 (63 atoms, 15×15×8 **k**-point grid), n=5 (99 atoms, 12×12×8 **k**-point grid), and n=6 (143 atoms, 10×10×8 **k**-point grid). For the interstitial, we use the following $n \times n \times 1$ supercells: n=4 (65 atoms, 16×16×8 **k**-point grid), n=5 (101 atoms, 12×12×8 **k**-point grid), and n=6 (145 atoms, 10×10×8 **k**-point grid). For the Frenkel pair, we use the following $n \times n \times 1$ supercells: n=5 (100 atoms, 12×12×8 **k**-point grid), n=6 (144 atoms, 12×12×8 **k**-point grid), and n=7 (196 atoms, 8×8×8 **k**-point grid). The relaxed structures are shown in Fig. S3.

Diamond: we use a cubic 8-atom unit cell with an experimental lattice constant of 3.567 Å. The **k**-point grid for the unit cell is 10×10×10. For the vacancy, we use the following $n \times n \times n$ supercells: n=2 (63 atoms, 5×5×5 **k**-point grid), n=3 (215 atoms, 4×4×4 **k**-point grid), and n=4 (511 atoms, 4×4×4 **k**-point grid). For the interstitial, we use the following $n \times n \times n$ supercells: n=2 (65 atoms, 5×5×5 **k**-point grid), n=3 (217 atoms, 4×4×4 **k**-point grid), and n=4 (513 atoms, 4×4×4 **k**-point grid). For the Frenkel pair, we use the following $n \times n \times n$ supercells: n=2 (64 atoms, 5×5×5 **k**-point grid), n=3 (216 atoms, 4×4×4 **k**-point

grid), and n=4 (512 atoms, 4×4×4 **k**-point grid). The relaxed structures are shown in Fig. S4.

Silicon: we use a cubic 8-atom unit cell with an experimental lattice constant of 5.43 Å. The **k**-point grid for the unit cell is 10×10×10. For the vacancy, we use the following $n \times n \times n$ supercells: n=2 (63 atoms, 5×5×5 **k**-point grid), n=3 (215 atoms, 4×4×4 **k**-point grid), and n=4 (511 atoms, 3×3×3 **k**-point grid). For the interstitial, we use the following $n \times n \times n$ supercells: n=2 (65 atoms, 6×6×6 **k**-point grid), n=3 (217 atoms, 4×4×4 **k**-point grid), and n=4 (513 atoms, 3×3×3 **k**-point grid). For the Frenkel pair, we use the following $n \times n \times n$ supercells: n=2 (64 atoms, 5×5×5 **k**-point grid), n=3 (216 atoms, 4×4×4 **k**-point grid), and n=4 (512 atoms, 3×3×3 **k**-point grid). The relaxed structures are shown in Fig. S5.

Tungsten: we use a body-centered cubic 2-atom unit cell with a converged lattice constant of 3.19 Å. The **k**-point grid for the unit cell is 30×30×30. For the vacancy, we use the following $n \times n \times n$ supercells: n=3 (53 atoms, 10×10×10 **k**-point grid), n=4 (127 atoms, 5×5×5 **k**-point grid), and n=5 (249 atoms, 2×2×2 **k**-point grid). For the interstitial, we use the following $n \times n \times n$ supercells: n=3 (55 atoms, 10×10×10 **k**-point grid), n=4 (129 atoms, 5×5×5 **k**-point grid), and n=5 (251 atoms, 2×2×2 **k**-point grid). The formation energy of the isolated Frenkel pair is obtained using these vacancy and interstitial calculations. For the unstable Frenkel pair, a 3×3×3 supercell with 54 atoms is employed, together with a 10×10×10 **k**-point grid. The relaxed structures are shown in Fig. S6.

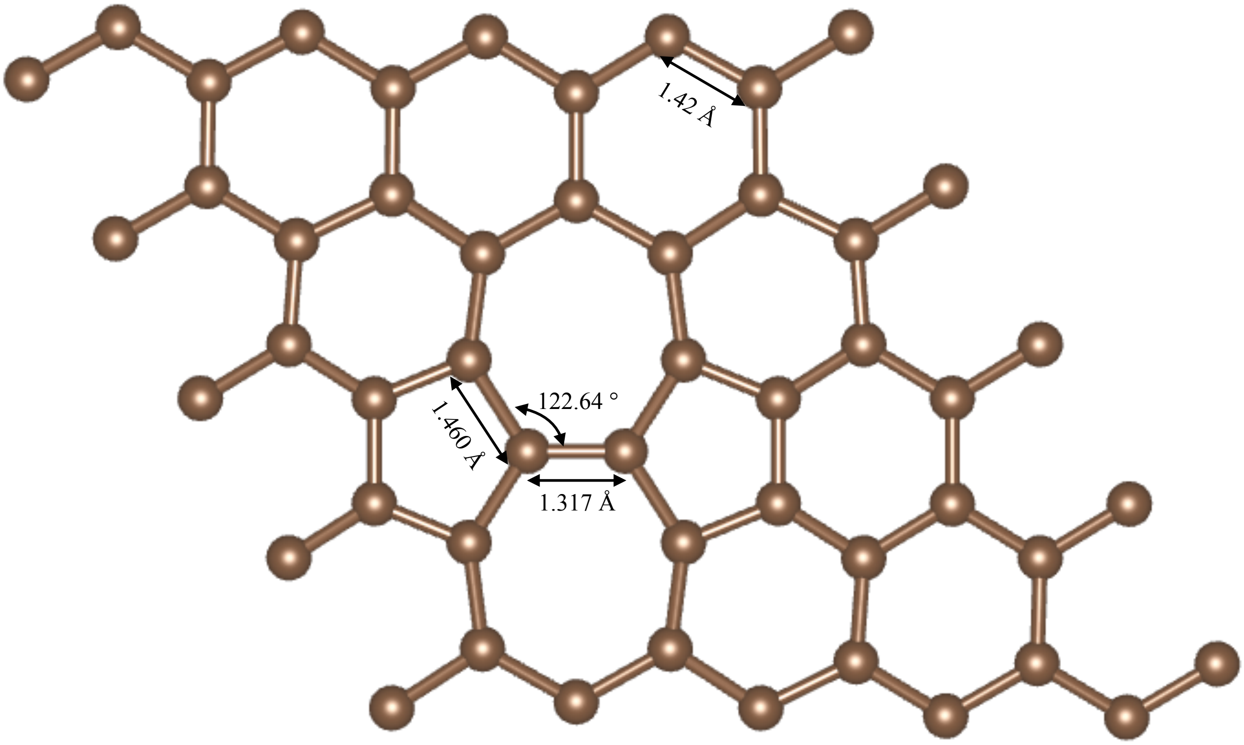

Figure S2. Stone-Wales defect in graphene. Shown is the relaxed structure of graphene with a SW defect. The bond lengths and angles around the SW defect are also shown, together with the C−C bond length of 1.42 Å in pristine graphene.

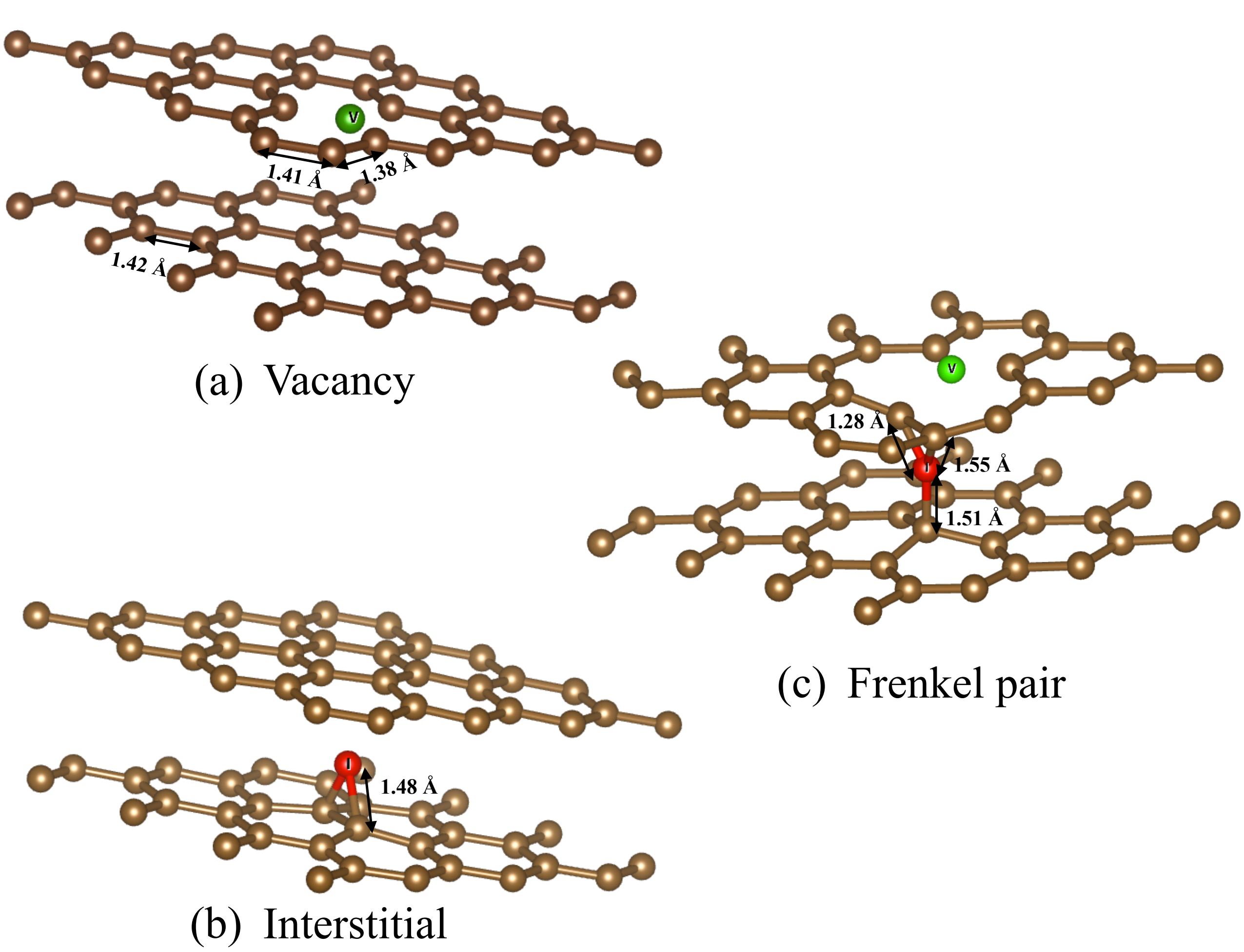

Figure S3. Defect configurations for graphite. Shown are the relaxed structures of graphite with a vacancy (**a**), an interstitial defect (**b**), and a Frenkel pair (**c**). The bond lengths around the defects are also shown. The distance between the vacancy and interstitial atom in the Frenkel pair is 2.33 Å. Green atoms marked with the letter V indicate vacancies and red atoms marked with the letter I indicate interstitials.

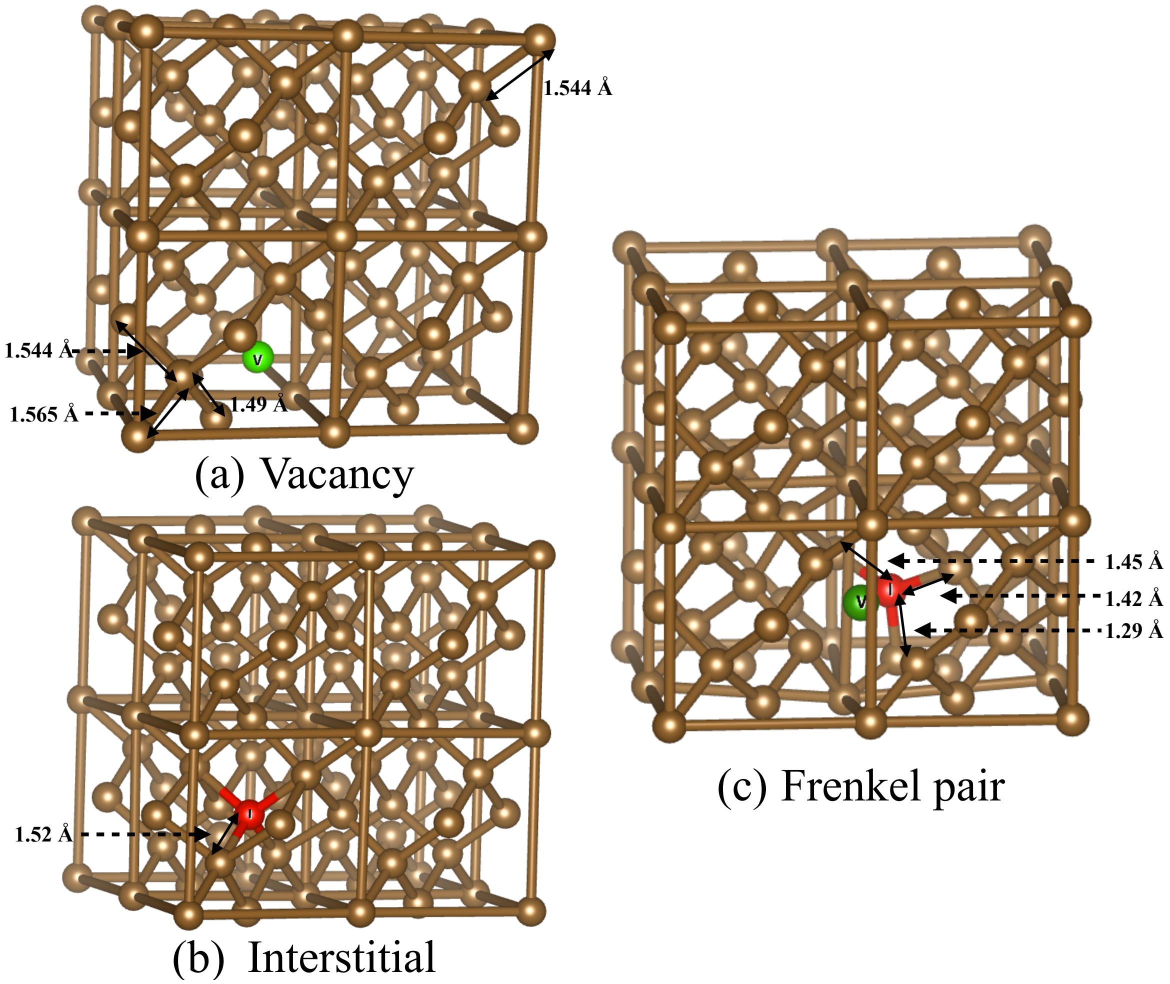

Figure S4. Defect configurations for diamond. Shown are the relaxed structures of diamond with a vacancy (**a**), an interstitial defect (**b**), and a Frenkel pair (**c**). The bond lengths around the defects are also shown. The distance between the vacancy and interstitial atom in the Frenkel pair is 2.58 Å. Green atoms marked with the letter V indicate vacancies and red atoms marked with the letter I indicate interstitials.

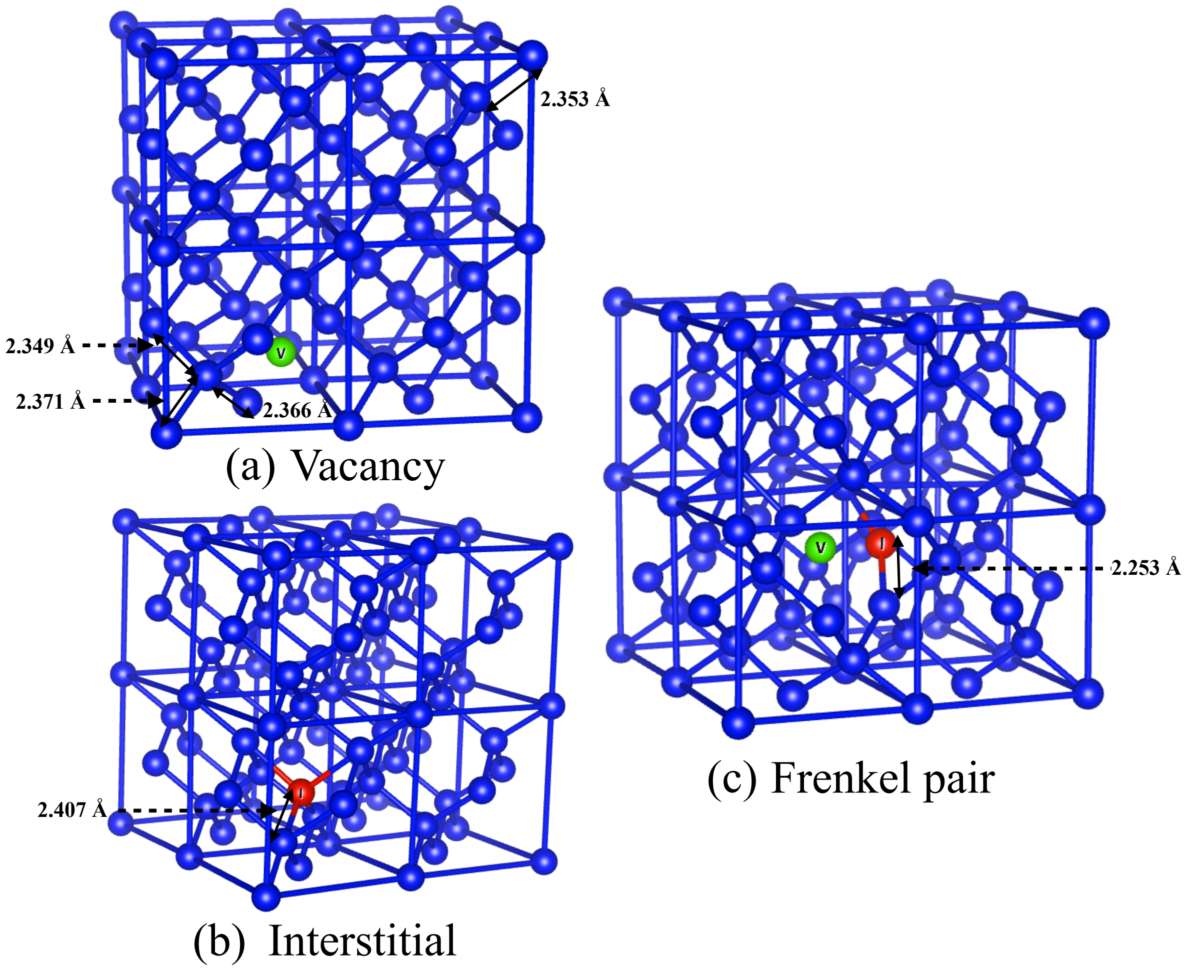

Figure S5. Defect configurations for silicon. Shown are the relaxed structures of silicon with a vacancy (**a**), an interstitial defect (**b**), and a Frenkel pair (**c**). The bond lengths around the defects are also shown. The distance between the vacancy and interstitial atom in the Frenkel pair is 1.93 Å. Green atoms marked with the letter V indicate vacancies and red atoms marked with the letter I indicate interstitials.

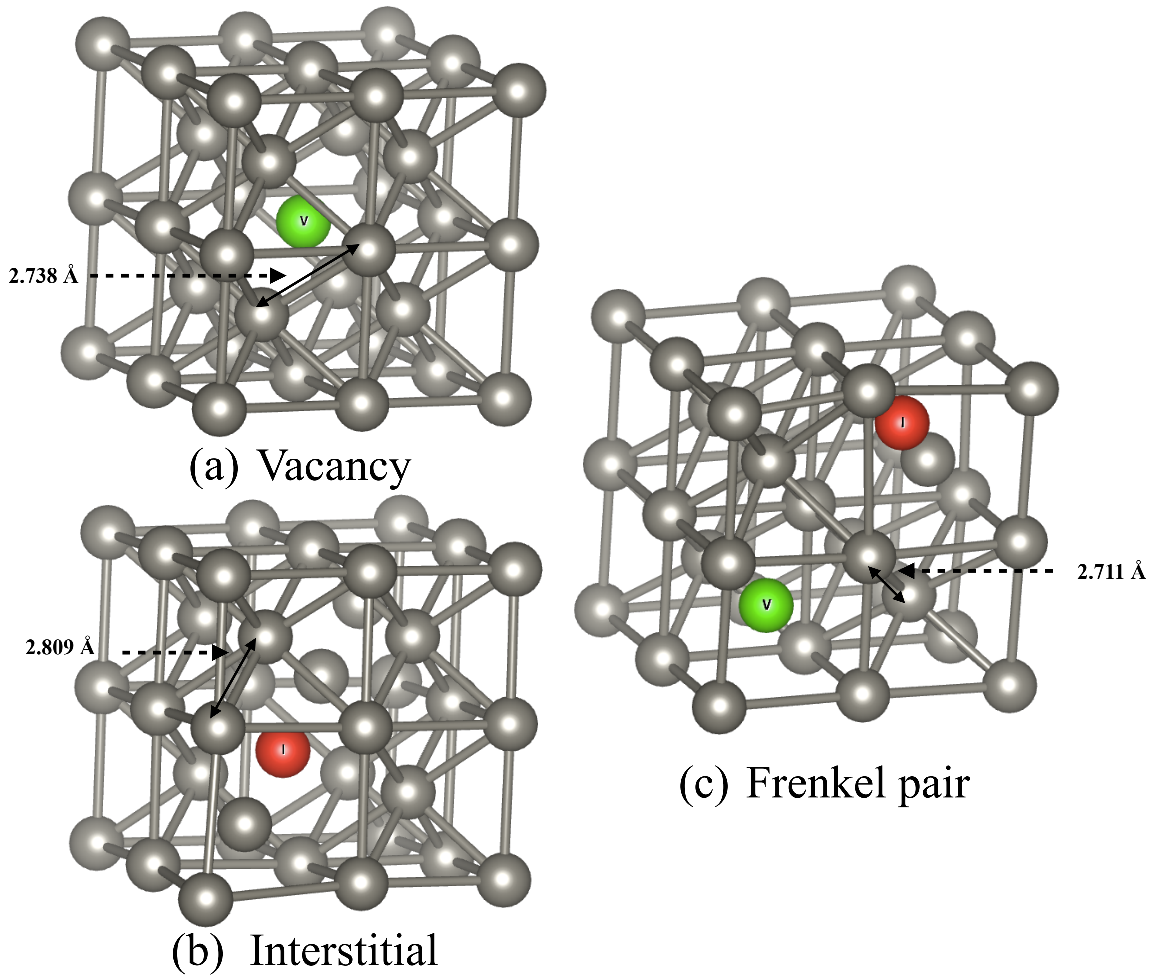

Figure S6. Defect configurations for tungsten. Shown are the relaxed structures of tungsten with a vacancy (**a**) and an interstitial defect (**b**). The bond lengths around the defects are also shown. The Frenkel pair shown in (**c**) is unstable and recombines in the DFT relaxation. For this reason, the isolated Frenkel pair formation energy is employed in this work. The distance between the vacancy and interstitial atom in the Frenkel pair is 4.82 Å. Green atoms marked with the letter V indicate vacancies and red atoms marked with the letter I indicate interstitials.

Fig. S7 shows the defect formation energy computed for supercells with increasing numbers of atoms, together with the extrapolated $N \to \infty$ values, which are also given in Table S1. These extrapolated formation energies are in very good agreement (within 5−10 %) with available experiments and previous calculations (Table S2). We find vacancy formation energies in the 4−8 eV range for the covalently bonded materials studied here, which are greater than the typical 0.5−4 eV values for metals[3]; for example, the vacancy formation energy computed here for tungsten is ∼3 eV. Our computed interstitial formation energies span a wide range, with values of 3−21 eV. The FP formation energy also varies widely (4.5−17.5 eV) for the materials studied here. In certain metals, FPs are stable against recombination only if the interstitial is at a minimum distance away from the vacancy[4,5]. For instance, the FP in tungsten is not stable[5] in our simulation cell and recombines upon DFT relaxation. For this reason, the FP formation energy in tungsten is estimated using the value for the IFP, namely, the sum of the vacancy and interstitial formation energies. We use the same approach to obtain E_{FP} in other materials for which we used computed FP formation energies taken from the literature (Table S3). The defect formation energies computed here, together with values taken from the literature for materials not studied here with DFT (Table S3), form the basis to compute the energy stored in defects.

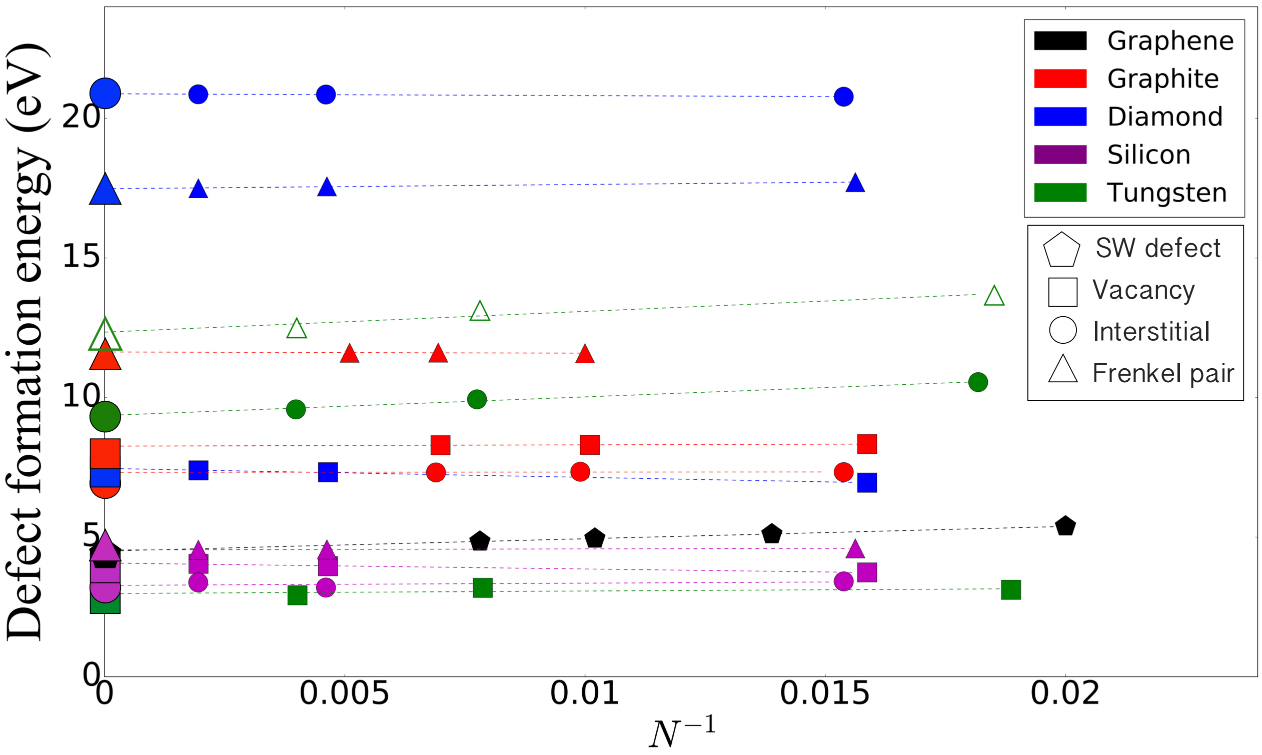

Figure S7. Defect formation energies computed with DFT for different types of defects considered in this work, plotted against the inverse of the number of atoms in the simulation cell. Different colors and shapes indicate different materials and defect types, respectively. The points on the y-axis are the extrapolated formation energies, employed in this work to compute the stored energy density.

Table S1. Converged defect formation energy, $E_F(N\rightarrow\infty)$ (units: eV)

Material	Defect	E_F	Material	Defect	E_F
graphite	V	8.26	silicon	V	4.07
	I	7.37		I	3.27
	FP	11.63		FP	4.54
diamond	V	7.46	tungsten	V	2.98
	I	20.88		I	9.35
	FP	17.48		FP	12.33[a]
graphene	SW	4.50			

V: vacancy; I: interstitial; FP: Frenkel pair; SW: Stone-Wales

[a] The value is taken to be the sum of the converged formation energy of a vacancy and an interstitial.

Table S2. Comparison of our computed defect formation energies with previous calculations and experiments. The number of atoms in the simulation cell is provided in parentheses for calculations taken from the literature that did not extrapolate the formation energy to the infinite number of atoms limit. Where this extrapolation was done, as in our work, an infinity symbol is put in parentheses. Units of eV are used throughout the table.

Material	Defect	Our E_F (number of atoms)	Available data (number of atoms)
graphene	SW	4.50 (∞)	4.66[6](∞)
graphite	V	8.33 (63), 8.30 (99), 8.29 (143), 8.26 (∞)	8.20[7](63), 7.6[8](53), 7.0[9](e)
	I	7.33 (65), 7.35 (101), 7.32 (145), 7.37 (∞)	7.8[8](55)
	FP	11.59 (100), 11.61 (144), 11.61 (196), 11.63 (∞)	11.6[10](109)
diamond	V	6.95 (63), 7.32 (215), 7.39 (511), 7.46 (∞)	7.2[11](31)
	I	20.78 (65), 20.86 (217), 20.86 (513), 20.88 (∞)	23.6[11](33)
	FP	17.72 (64), 17.57 (216), 17.50 (512), 17.48 (∞)	16.5–19[12](216)
silicon	V	3.74 (63), 3.96 (215), 4.04 (511), 4.07 (∞)	4.1[13](63), 3.56[14](216), 3.3[15](216), 4.0[16](e)
	I	3.42 (65), 3.20 (217), 3.38 (513), 3.27 (∞)	3.31[17](128), 3.3[13](65), 3.56[18](e)
	FP	4.60 (64), 4.56 (216), 4.55 (512), 4.54 (∞)	4.26[14](216)
tungsten	V	3.11 (53), 3.18 (127), 2.918 (249), 2.98 (∞)	3.56[19](127), 3.11[20](128), 4.0[21](e)
	I	10.55 (55), 9.94 (129), 9.58 (251), 9.35 (∞)	9.55[19](129), 9.98[5](251), 9.82[20](250), 9.06±0.63[22](e)

Note: SW: Stone-Wales defect; V: vacancy; I: interstitial; FP: Frenkel pair. (e): experimental data.

Table S3. Vacancy and interstitial formation energies in materials for which first-principles calculations were not carried out in this Work. The data are taken from DFT calculations found in the literature, and are employed in Fig. 3 of the main text for the materials shown with blue squares and labeled as "available calculations". The isolated Frenkel pair formation energy is obtained here as the sum of the vacancy and interstitial formation energies. Units of eV are used throughout the table.

Material	Vacancy	Interstitial	Isolated Frenkel pair
Be	1.09[4]	4.01[4]	5.10
Al	0.58[23]	1.58[24]	2.16
Ti	1.97[25]	2.13[25]	4.10
V	2.51[26]	3.14[27]	5.65
Cr	2.64[26]	5.66[26]	8.30
Fe	2.02[28]	3.75[29]	5.77
Mo	2.96[30]	7.34[27]	10.3

[1] Kittel, C. *Introduction to Solid State Physics* (Wiley, 2005).

[2] Angsten, T., Mayeshiba, T., Wu, H. & Morgan, D. Elemental vacancy diffusion database from high-throughput first-principles calculations for fcc and hcp structures. *New J. Phys.* **16**, 015018 (2014).

[3] Dudarev, S. Density functional theory models for radiation damage. *Annu. Rev. Mater. Res.* **43**, 35–61 (2013).

[4] Middleburgh, S. & Grimes, R. Defects and transport processes in beryllium. *Acta Mater.* **59**, 7095–7103 (2011).

[5] Ahlgren, T., Heinola, K., Juslin, N. & Kuronen, A. Bond-order potential for point and extended defect simulations in tungsten. *J. Appl. Phys.* **107**, 033516 (2010).

[6] Shirodkar, S. N. & Waghmare, U. V. Electronic and vibrational signatures of Stone-Wales defects in graphene: First-principles analysis. *Phys. Rev. B* **86**, 165401 (2012).

[7] Telling, R. H., Ewels, C. P., El-Barbary, A. A. & Heggie, M. I. Wigner defects bridge the graphite gap. *Nat. Mater.* **2**, 333–337 (2003).

[8] Li, L., Reich, S. & Robertson, J. Defect energies of graphite: Density-functional calculations. *Phys. Rev. B* **72**, 184109 (2005).

[9] Thrower, P. A. & Mayer, R. M. Point defects and self-diffusion in graphite. *Phys. Status Solidi A* **47**, 11–37 (1978).

[10] Yazyev, O. V., Tavernelli, I., Rothlisberger, U. & Helm, L. Early stages of radiation damage in graphite and carbon nanostructures: A first-principles molecular dynamics study. *Phys. Rev. B* **75**, 115418 (2007).

[11] Bernholc, J., Antonelli, A., Del Sole, T. M., Bar-Yam, Y. & Pantelides, S. T. Mechanism of self-diffusion in diamond. *Phys. Rev. Lett.* **61**, 2689–2692 (1988).

[12] Goss, J. P., Rayson, M. J., Briddon, P. R. & Baker, J. M. Metastable Frenkel pairs and the W11–W14 electron paramagnetic resonance centers in diamond. *Phys. Rev. B* **76**, 045203 (2007).

[13] Blöchl, P. *et al.* First-principles calculations of self-diffusion constants in silicon. *Phys. Rev. Lett.* **70**, 2435–2438 (1993).

[14] Goedecker, S., Deutsch, T. & Billard, L. A fourfold coordinated point defect in silicon. *Phys.*

Rev. Lett. **88**, 235501 (2002).

[15] Puska, M., Pöykkö, S., Pesola, M. & Nieminen, R. Convergence of supercell calculations for point defects in semiconductors: Vacancy in silicon. *Phys. Rev. B* **58**, 1318–1325 (1998).

[16] Fukata, N., Kasuya, A. & Suezawa, M. Formation energy of vacancy in silicon determined by a new quenching method. *Physica B* **308-310**, 1125–1128 (2001).

[17] Leung, W.-K., Needs, R., Rajagopal, G., Itoh, S. & Ihara, S. Calculations of Silicon Self-Interstitial Defects. *Phys. Rev. Lett.* **83**, 2351–2354 (1999).

[18] Kamiyama, E., Sueoka, K. & Vanhellemont, J. Formation Energy of Intrinsic Point Defects in Si and Ge and Implications for Ge Crystal Growth. *ECS J. Solid State Sci. Technol.* **2**, P104–P109 (2013).

[19] Nguyen-Manh, D., Horsfield, A. P. & Dudarev, S. L. Self-interstitial atom defects in bcc transition metals: Group-specific trends. *Phys. Rev. B* **73**, 020101 (2006).

[20] Becquart, C. & Domain, C. Ab initio calculations about intrinsic point defects and He in W. *Nucl. Instr. Meth. Phys. Res. B* **255**, 23–26 (2007).

[21] Maier, K., Peo, M., Saile, B., Schaefer, H. E. & Seeger, A. High–temperature positron annihilation and vacancy formation in refractory metals. *Philos. Mag. A* **40**, 701–728 (1979).

[22] Neklyudov, I. M. *et al.* Interstitial atoms in tungsten: Interaction with free surface and in situ determination of formation energy. *Phys. Rev. B* **78**, 115418 (2008).

[23] Bottin, F. & Zérah, G. Formation enthalpies of monovacancies in aluminum and gold under the condition of intense laser irradiation. *Phys. Rev. B* **75**, 174114 (2007).

[24] Jesson, B. J., Foley, M. & Madden, P. A. Thermal properties of the self-interstitial in aluminum: An ab initio molecular-dynamics study. *Phys. Rev. B* **55**, 4941–4946 (1997).

[25] Tunde Raji, A. *et al.* Pseudopotential study of vacancies and self-interstitials in hcp titanium. *Philos. Mag.* **89**, 1629–1645 (2009).

[26] Derlet, P. M., Nguyen-Manh, D. & Dudarev, S. L. Multiscale modeling of crowdion and vacancy defects in body-centered-cubic transition metals. *Phys. Rev. B* **76**, 054107 (2007).

[27] Han, S., Zepeda-Ruiz, L. A., Ackland, G. J., Car, R. & Srolovitz, D. J. Self-interstitials in V and Mo. *Phys. Rev. B* **66**, 220101 (2002).

[28] Domain, C. & Becquart, C. S. Ab initio calculations of defects in Fe and dilute Fe-Cu alloys. *Phys. Rev. B* **65**, 024103 (2001).

[29] Willaime, F., Fu, C., Marinica, M. & Dalla Torre, J. Stability and mobility of self-interstitials

and small interstitial clusters in α-iron: ab initio and empirical potential calculations. *Nucl. Instr. Meth. Phys. Res. B* **228**, 92–99 (2005).

[30] Mattsson, T. R. & Mattsson, A. E. Calculating the vacancy formation energy in metals: Pt, Pd, and Mo. *Phys. Rev. B* **66**, 214110 (2002).
